# Medicine and Its Achievements

**Published:** 1904-08

**Authors:** 


### SELECTIONS AND ABSTRACTS.

### Medicine and Its Achievements.*

As provided by the Constitution and By-Laws of the Louisiana State Medical Society, it is the duty of the President to deliver an address at some time during the annual session on some medical or kindred subject. And indeed it has been duty only that has forced me to make the attempt, for the pleasure of this occasion has long since evaporated in the preparation of this infusion.

It is not my desire that it shall have an hypnotic effect, but should you discover your eyes becoming dim and you falling in the arms of Morpheus I will most willingly excuse you, for one at home more interested in me than any one else lapsed into gentle slumber under its reading.

It is with no little embarrassment that I appear before you when I remember that I am the twenty-fifth president of the Society, and my predecessors have been men learned in the arts, sciences and literature, most of them professors and teachers of medicine of national reputation, and some orators of no mean repute. I can not expect more than that this address will be but a cheap imitation of the model set for me by my distinguished predecessors, and out of charity and generosity I would ask you to make no comparison.

I have been hampered in the selection of a subject. To select one with which I was familiar, you know more about it than I; and could I choose one of which you knew nothing, I would know less.

But as this is a public occasion, and expecting a number of the laity to honor us with their presence, I have chosen for the subject of my address "Medicine and Its Achievements," in the hope that it might in some degree interest them; while to the members of the Society nothing new can be presented, but as the recital of

* Annual Address of President, read by J. M. Barrier, M.D., of Delhi, La., at the meeting of the Louisiana State Medical Society, May 10, 11, 12, 1904.

deeds of valor and heroism in the defense of country kindles anew our patriotism, so the recounting of the thoughts and discoveries of medicine may incur our zeal and love for our calling and be a stimulus to press onward to still greater achievements.

The history of medicine had its origin with that of the human race, and its progress and development has been proportionate to that of human knowledge and human civilization.

The foundation of medicine lies deep in that soil of human knowledge from which arose all the sciences. As we take an extended view of its progress, we can trace it from its scanty sources in the most remote periods of society, augmented by the stores of Roman and Grecian literature, obscured by the darkness of the middle ages, and finally bursting forth in the copious and majestic stream of modern medicine. From its beginning to the present this stream has been fed by the rivulets of all the sciences, and in time it has freely contributed to them all and has added to all the arts of life and to the gradual progress of knowledge on all subjects connected with our existence and welfare. Disease is the heritage of humanity and death is the common lot of all; and man has ever been trying to relieve the former, and delay the inevitability of the latter.

A system of medicine and surgery has existed in every age and every clime; crude it may have been in the barbarous state and approaching a more rational system with man's own progress in knowledge and civilization.

The historical records of medicine among the ancients are scanty and meagre. Egypt was the first country in which the art of medicine was cultivated with any degree of success and had advanced so far as to become a distinct profession. It is probable that the priests of the Egyptians were the physicians, and indeed this seems to have been the natural process of society, in its earlier periods, when the priests were the depositories of knowledge of all sorts, and when they confined it as much as possible to their own use, for the purpose of maintaining their influence in the community. While among the Egyptians medicine had become a distinct profession, the system consisted in great part in the employment of incantations in magic. This has been in all cases the first step in the art of medicine, and its efficacy has been dependent upon the ignorance and superstition of the people among whom it was exercised.

Assyria may contest the claim with Egypt for the parentage of the arts and sciences, but their memorials are so scanty that it is conjectural as to what degree of excellence they had arrived. The priests of this nation, as in Egypt, possessed all the knowledge of medicine, which consisted of little more than the dexterous application of the arts of magic and such other means as tended to impress the people with a sense of their power over the operations of nature.

From Egypt we can trace the progress of medicine into Greece, in the remote and semi-fabulous ages of their demigods and heroes until it acquired the rank of science under the genius of Hippocrates. It is curious to note that with the introduction of medicine in Greece its progress is identified with the life and history of some great name. The first worthy of consideration is the name of Æsculapius, to whom is ascribed the merit of having devoted himself to the cultivation of medicine as a science and having made it a distinct object of pursuit. In such esteem was he held by his countrymen that, after death, divine honors were paid him. He was designated the "God of Physic" and temples were erected to him in various parts of Greece. And to this day all practitioners of medicine are called the "sons of Æsculapius."

The practice of medicine remained a considerable time hereditary in the family of Æsculapius, and his descendants received the name of Æsclepiadæ—they were priests of the temple, and these temples became a species of hospitals to which patients resorted from all quarters. While certain practices of a dietetic nature were enjoined, and measures conducive to temperance and cleanliness were practiced, and internal remedies were given, the priests of these temples relied in most part on magic and incantations, and sedulously kept up the system of rites and ceremonies which had been handed down to them from still more ancient practitioners. They carefully preserved the sole management of the art over which they presided. The symptoms were recorded on votive tablets and these half-priestly and half-physiologic caste of Æsclepiadæ compiled the data upon which the earliest generalizations of medicine as an inductive science was based. But the causes of the morbid state and the rational of treatment was not sought for. "The anger of a God was sufficient reason for the existence of a malady and a dream was ample warranty for therapeutic measures; that a physical phenomenon must needs have a physical cause was not the implied or expressed axiom as it is in this scientific age."

Centuries elapsed and the practice of medicine remained almost stationary in the hands of the priesthood.

About the sixth century before the Christian era a spirit of improvement took place, and the genuine principles of philosophy made their appearance in Greece, and, among other topics, the powers and functions of the human body, the nature and cause of disease and means for their control.

Pythagoras may be mentioned as the first to give an impetus to the study of the various natural sciences. He is supposed to have dissected the bodies of animals, acquiring a certain acquaintanee with anatomy, and gaining some knowledge of the structure and action of the human frame. After Pythagoras few contributed anything to improve medicine, until the arrival of the justly celebrated Hippocrates, who received from his contemporaries the title of "Father of Medicine," which has ever since remained. He effected a complete revolution in the practice and profession of medicine and introduced a system which may be considered as having laid the foundation for all its future improvements, and it may be confidently affirmed that the science of medicine is more indebted to him than to any single individual.

After Hippocrates, for nearly six centuries, there appeared no great name associated with medicine. About 300 years before Christ the School of Alexandria was established by the munificence of the Ptolemies, and who laid the foundation for the great Alexandrian Library. The science of medicine was cultivated in this school, and there for the first time was a human subject dissected, and we owe some very marked improvements to the professors of this school. This school for several centuries produced a succession of great men, who advanced knowledge and prevented the decay of science and literature after the decline of the Grecian Empire.

The Roman Empire was extending her dominion and was laying the foundation of her future greatness and was given almost exclusively to war. Science of all kinds was neglected, but especially was this true of medicine. Pliny tells us that for six hundred years Rome had no physicians, and in the language of Voltaire, "Rome for five hundred years had no physicians—their occupation was slaughter, and not the art of prolonging life." The first physicians in Rome were slaves. The celebrated Musee, physician to Augusta, was a slave who was freed and elevated to nobility. In the first century after the Christian era, there appeared Celsus, the Roman Hippocrates, contemporaneous with Virgil and Cicero. He is the first native Roman physician whose name has been transmitted to us. He is also the first writer to treat on surgery and surgical operations, and some of the capital operations seemed to be well understood and frequently practiced.

In the next century there appeared Galen, the physician of Marcus Aurelius, and from his marvelous speech and healing was called the "Wonder Worker," and of whom a recent writer said: "He acquired a name which for centuries was above every name, and even now stands pre-eminently illustrious."

Then came the "dark ages," that period of eclipse when human progress seemed to have come to an end, when all advancement in science, art and literature seemed doomed. Superstition and ignorance had taken possession of the whole civilized world. Rome had been overrun by the northern barbarians and lost her former glory and power. Medicine, along with all science and knowledge, suffered the same fate, and for centuries there appeared but few names to shed even a ray of light in this dark and benighted period. To the monk, hidden away in his cloister, we are indebted for the preservation of medicine, "uniting in himself the twin office of intercessor with God and the healer of men."

This period of darkness seemed to be eternal, but at last the dawn of day was seen to be breaking, and with the revival of letters a new impetus was given to the pursuit of all sciences—medicine among them. In the sixteenth century there appeared Vesalius, the first anatomist, who we may justly say laid the foundation for modern medicine. From that time till the present what a galaxy! Harvey, Hunter, Sydenham, Jenner, Morton, Simpson, McDowell, Sims, Virchow, Lister, Koch, Pasteur, and many others whose names will live as long as a civilized tongue is spoken, or as long as humanity is heir to death and disease.

### THE ACHIEVEMENTS OF MEDICINE.

In the early morn of the twentieth century we behold a civilization before which Grecian splendor and Roman magnificence pale into utter insignificance; distance annihilated, the inhabitants of the remotest corners of the earth are near neighbors; the future is compressed into the present by the telegraph and the telephone; magnificent steamers plough the turbulent seas as easily as the crude dug-out the placid and peaceful streams of the interior; intelligence and education well-nigh universal; the poor have the comforts of life; the workingman has luxuries that even princes a century ago could not afford; commerce has rendered possible the exchange of every country and every nation.

What part has medicine played in this great drama? What has medicine contributed to human progress and man's physical, mental and moral betterment?

As a social factor it centers into every relation of human life, and as an economic factor there is no calling or pursuit that escapes its influence.

The greatest achievement of medicine is the evolution of a rational and scientific system of practical medicine. Medicine has ever been permeated with empiricism and dogma, and has had to contend with ignorance and superstition. In the discovery of new truths and in the enactment of every measure for the benefit of mankind and in every effort for its own improvement, it has met the opposition of the selfish politician and the superstition and incredulity of an ignorant public. Theory alone governed medical men for ages. It made an early effort to free itself. The most brilliant epoch of Grecian history is marked no more immortally by the wisdom of Socrates, the histories of Herodotus, the tragedies of Achilles and the art of Phidias, than by the medicine of Hippocrates. This period represents the first endeavor to break away from the empiricism of the early ages. From that time on we find science and empiricism ever contending. The struggle still continues, but it is an unequal contest and science is sure to be victorious. At no period of the world's history has scientific medicine been so aggressive, and in the beginning of the twentieth century the victory has been almost achieved. We see the vagaries of Hahnemann, the charlatanism and humbug of osteopathy and Christian Science, along with the "isms" and "pathics," gradually dissolving before the searchlight of truth and science. We will soon behold a school of medicine having for its foundation truth and science—the members of which, wearing no badge of sectarianism, whose motto is to be proclaimed by its noble deeds and altruistic life *in the service of humanity.*

The primary object of medicine is the relief of pain and the cure and prevention of disease. Has it accomplished the first? The whole civilized world, with one loud voice, acclaim "*Yes.*" The fool has said in his heart, "There is no God;" the fool has said in his heart, "There is no good in medicine"—but racked with pain and fears of impending dissolution, his infidelity vanishes, and he, too, cries, "Go for the doctor, and tell him to come quick!"

Has medicine cured disease? While death is inevitable and the cemetery has its short graves and its long ones, no one is so skeptical as to deny, and under the sound of my voice there are grateful hearts that attest that their lives have been saved, a wife, a mother, been spared to loved ones by the watchful care and skill of some physician. The profession of medicine has sometimes been taunted with its failures, but can you mention any calling or profession without its failures and mistakes? The legal profession boasts of its learning, and its enduring monument is the great men it has produced. In every case that is tried before judge or jury there are two sides, and one side loses every time. Was there ever a law so enacted that some shrewd lawyer could not find a flaw? The Supreme Court of the United States, the most learned judicial tribunal in the world, seldom gives a unanimous opinion. The majority rules, and yet the dissenting judge is just as learned. With every high water some distinguished engineer makes an inspection of the levees, and the papers herald the fact that "Major So-and-So has made a thorough inspection of the levees and pronounced them safe and secure, and would stand." And yet the flood comes and some poor fellow sees the result of the labors of a lifetime making a "bee-line" for the Gulf of Mexico. A short time ago the inhabitants of St. Pierre, Martinique, heard a rumbling sound from an extinct volcano; the people were alarmed, and a learned man of science was sent to make an examination and he reported all safe—"No danger." The world knows the consequences.

Comparing the results of medicine with other callings, the profession of medicine comes as near accomplishing its objects and purposes as any other.

By the discovery of antitoxin for diphtheria the mortality has been reduced in this disease from about 40 to 7 per cent. In Chicago, between October 5, 1897, and December 31, 1903, there were treated 7,435 cases with antitoxin, with 479 deaths—about 6½ per cent. Without antitoxin the percentage was 35.

Typhoid fever, one of the most prevalent diseases and constantly present in all centers of population, under improved treatment has been reduced in twenty-five years in the hospitals of the world from 20 to 35 per cent. to from 5 to 15 per cent. in its death rate. The average mortality of all diseases in the last fifty years has been reduced about fifty per cent. The discovery of anesthesia, in 1846, marks a most important epoch in history. If medicine had never made another discovery this alone would entitle it to the world's gratitude and homage. How many lives it has saved eternity alone will reveal. It has annulled the penalty placed on woman, "in travail shalt thou bring forth the young." Chloroform has reduced the mortality in obstetrics more than all other means.

Anesthesia has helped mightily to develop surgery into its present state of perfection. As Americans, we may justly feel proud of our surgeons. It was an American who first performed ovariotomy; that has alone saved the lives of thousands of women. It is estimated that this operation has added 40,000 years to the lives of women in Great Britain alone and other countries in proportion. It was an American, Marion Sims, who devised and first performed an operation which is now practiced throughout the civilized world. Woman's great benefactor—"his name will be spoken in every tongue where woman suffers and man sympathizes."

It is in preventive medicine that the greatest victories have been achieved. The noblest aim of the physician is to prevent disease. In this age of commercialism there are men racking their brains to make a discovery or an invention to be patented for their own exclusive pecuniary reward; there are men spending their lives immolating self in the seclusion of the laboratory to discover something to exterminate disease and to be given to the world.

Beginning with the immortal Jenner and vaccination for the prevention of smallpox, down to the present, what contributions have been made to the world by the science of medicine in preventing disease! What has vaccination done for the world? It has defeated in open conflict one of the deadliest enemies of mankind, and has put us upon lines of investigation which may yet rob all pathogenic organisms of their terrors. Before the introduction of vaccination smallpox at times would almost decimate the whole of Europe. It would seem that no argument would be necessary to prove the efficacy of vaccination, yet there are some who have no faith in it—even antagonizing it. The discovery of vaccination and its results is among the greatest achievements of any age, and it stamps Jenner as among the world's greatest benefactors. When the names of Hannibal and Napoleon shall have been forgotten, Edward Jenner will live on and on, and as years are added to years, there will be added glory and luster to his name.

The finding of the mosquito as the cause of malaria and yellow fever must be recorded among the greatest discoveries of the nineteenth and twentieth centuries. This will change the geography of the world. It may never completely eradicate these diseases, but marvelous results have already been accomplished. In Havana, in 1901, reports showed that there were 20,000 different premises containing mosquito larvæ, and some time ago a similar investigation showed but 200. Havana has been literally converted into a health resort. A short time ago Major Gorgas reported that Havana was absolutely free from yellow fever, and would remain so, and in his words, "the sanitary rehabilitation of Havana is a monument to American science and energy." What has been accomplished in Havana has likewise been done in Freetown, in Africa, under British dominion. To make more impressive the importance of the discovery of the mosquito as the cause of malaria, Dr. Gardiner, in his address before the British Association, says: "Malaria, of all diseases of our tropical possessions, is the most important. It undermines the health of millions, and makes vast regions which would otherwise be our richest possessions almost uninhabitable. Every year in India alone it kills some 5,000,000 people—twice as many as cholera, smallpox, plague and other infectious diseases put together." Says another, speaking of the malarial germ: "It has played a greater part in human affairs than the greatest general or politician who ever lived. There will be added to the commerce of the world millions and the richest and most fertile regions of the globe will be opened to the Anglo-Saxon and his civilization. The discovery of the mosquito as the cause of malaria and yellow fever, along with sanitary measures, will make it possible for the construction of the Panama canal, an engineering feat worthy of any age, and yet no member of the medical profession was worthy a place on the Canal Commission."

I might at length go into the details of the discoveries of the causes of many infectious diseases which have been arrived at by Koch, Klebs, Lœffler, Frankel and Laveran, and many others. The bacterial cause of tuberculosis, typhoid fever, diphtheria, pneumonia, erysipelas, gonorrhea, cerebro-spinal meningitis, dysentery, the plague, charbon, glanders and influenza has been discovered and proven, and as a result their spread has been lessened and their mortality lowered. The names of Virchow, Lister, Koch and Pasteur will be forever associated with the greatest and most brilliant achievements of medicine in the nineteenth century.

To ask again, what has medicine accomplished? Dr. Parker, of London, estimated that smallpox has diminished 95 per cent.; deaths from fevers generally have declined 82 per cent.; from typhus fever 90 per cent.: from enteric fever 60 per cent.; from scarlet fever 81 per cent.; from phthisis 46 per cent.

Mr. Edwin Shadwick, as far back as 1878, stated to the British Scientific Association that both the sick and death rate had been diminished one-third by the practice of sanitary measures, and since then it has been reduced more than one-half.

Says another, in more beautiful and eloquent language than I can command: "It has lengthened life, it has mitigated pain, it has extinguished disease. It has explored with amazing minuteness the mysteries of man's own being, has analyzed all the tissues of his body, and isolated the minutest and ultimate cells of which those tissues are composed, wherein are conducted the secret forces of those little cells that require the keen eye of the organs and systems of wonderful adaptation and design; and how in turn these organs and systems are combined to form the noblest work of God."

Led by Harvey it has discovered the vital fountain from which gushed crimson streams of blood that ramify and wind through all the labyrinths of the system, distributing life, warmth and food to its remotest parts. Guided by Laennec it has placed the ear to the chest and interpreted the vesicular murmur within where air and blood meet to exchange the gaseous elements with which they are freighted. Taught by that splendid mathematical genius of Henrique Helmholtz, it has sent luminous beams into the deepest and remotest recesses of the human eye, and revealing there a picture of bewitching beauty, has shown how disease may shut out those beams forever and leave all in eternal darkness, and how a steady and skillful hand reopening a darkened eye may achieve one of the greatest successes of the healing art. It has followed the waves of sound along the intricate paths they tread to sensitive chambers of audition, and learned the delicate mechanism of that acoustic apparatus, which can adjust itself to the faintest whisper, or protect itself against the loudest roar of artillery. Utilizing beautiful optical laws, it has illuminated the interior of the larynx and seen the precision and rapidity of that alternate advancing and receding tension and relaxation of the vocal cords which give to the human voice its tones of tender harmony or thrilling eloquence. And not content here it has ascended the dome of thought—the palace of the soul—and beheld the wonderful inhabitants of the royal mansion where dwells the king of all our thoughts, perceptions and volitions. Familiar with all these organs and systems when moving in harmonious order, that same science of medicine stands upon the watch tower and, like a faithful sentinel, sounds the first note of alarm at the approach of danger, and armed with the agents devised by its own fertility, is ever ready to send them forth on errands of succor. More, it has taught cunning to the hand of the surgeon to guide the keenest blade on its bloody, but efficacious mission, and like an angel of mercy is able in the sleep of anesthesia to rob that edge of its pain and anguish.

What the world owes the science of medicine in fighting disease can never be estimated, but as an economic factor in the world's present commercial greatness, it deserves consideration. It is doubtful whether the average citizen has ever associated medicine with anything but disease, and has ever for a moment placed a commercial value to it. Except when there is some acute disease for the physician to attend, by the average layman he is regarded as a drone in this hustling and bustling world. Were we to compute the commercial value of the profession of medicine to the State, the amount would astound mathematic computation. There are about 1,500 physicians in the State of Louisiana and estimating that they each saved yearly six human lives that would be 9,000 lives; putting a commercial value of $2,000 per life that would be $18,000,000. It is safe to say that the average income of the Louisiana physician is not more than $1,500, making $2,250,000—leaving a balance of $15,000,000 annually. Add to this amount the time saved by attention of the physician in shortening sickness and to pay the arrearage for the last twenty years would bankrupt the State. No other calling renders such services at such wages.

Commerce, agriculture, manufacturing, mining, and every commercial and industrial pursuit is indebted to medicine and its allied sciences.

This beautiful city now progressing along all lines by leaps and bounds, now the second largest port in America, and destined to be the largest in the world—owes more to the science of medicine than to any other factor or agency. After the terrible epidemic in 1878 this city was almost financially ruined, houses vacant, families decimated, loved ones gone—the cemeteries the abiding-place of some of your best, noblest and truest citizens; mourning had taken the place of mirth, the future of your city and this beautiful Southland seemed indeed dark. She arose from her "sackcloth and ashes" and to-day her beauty, glory and greatness eclipse any former period. What saved your city? A system of quarantine was established, the product of the brain of a Louisiana physician, which system has become a model for the civilized world. The name of Joseph Holt will live in the hearts and affections of this people. Not waiting to cast a flowery garland on his bier, I take this opportunity on behalf of this Society and the profession in the State of Louisiana to express the acknowledgments of gratitude and appreciation of his loving and admiring confreres.

The wheels of commerce would stop in twenty-four hours were it not for the science of medicine in originating, perfecting and operating the present system of maritime quarantine. The great steamships riding the waves, conquering the storms and tempests of the mighty ocean, distributing and exchanging the products of every clime, can not leave or enter port without its bill of health vised by a medical man. The architect may plan a building, but its sanitary arrangements must pass the inspection of medical science. No immigrant can enter the country without the stamp of medical approval. The army and navy owe their efficiency to the surgeons' examinations. The life insurance companies—the greatest commercial institutions of the world—owe their very existence to the science of medicine and to the honesty, skill and knowledge of their medical examiners. What the chemist has accomplished in the last one hundred years would read like a story from the Arabian Nights. While the triumphs of chemistry along synthetic lines have been great, the triumphs of the analytic chemist have been equally great. The analytic chemist is protection against the fraudulent products of the synthetic chemist. Inspection of the food and water supply in any community is absolutely essential and is one of the most important functions of every board of health. The stamp of the board of health on your meat has saved you of many a sickness and the analysis of the water supply has saved many a city from an epidemic of typhoid fever.

The animal industry owes its present growth to veterinary medicine. The discovery of anthrax virus has as completely swept away charbon as vaccination has smallpox.

The great intellectual growth of our country and the general interest taken in education has been aided by the profession of medicine. One of the fundamental objects of the American Medical Association was the elevation of the standard of the physician, and well has that object been accomplished in this country, for only a few decades ago our best medical colleges only required two courses of about four months each, and to-day all first-class medical colleges require a four years' course and each student is required to devote a certain time to laboratory work in bacteriology, pathology, chemistry and pharmacology. Not only has the standard in professional attainments been raised, but to enter the study of medicine an increased scientific and literary education is required. As the ultimate end of medicine is the prevention of disease and the enforcement of the strict observance of all sanitary laws, a people must be educated. An educated people is the strongest ally the profession has in enacting and enforcing sanitary measures. And to accomplish its high aims and purposes, contends for increased educational advantages for the people, knowing that sanitation and hygienic regulations can never be enforced on an ignorant and superstitious people.

The profession of medicine has ever stood for morality. No one knows better than the physician the evil effects physically from the violation of the moral code. The profession of medicine is the exponent of personal purity and morality. To the stay of the demon, strong drink, it has contributed the most important part. By the scientific investigation of the effects of alcohol, it has added common sense and reason to the temperance fanatic. The W. C. T. U., and God bless them for their noble work, can count the medical profession, as a whole, as their strongest ally in this great work.

The profession of medicine in all ages has ever stood for the true, the beautiful and good, and its contributions to the enlarged humanitarianism of the age is one of its grandest and noblest achievements. With a broad spirit of catholicity it knows no creed, no sect, no race! Its ear is open to the cry of humanity. "Am I my brother's keeper?" went reverberating down the ages for 4,000 years, with no answer, save the echo and re-echo of its own question, until the "Star of Bethlehem" stood over the little babe in the manger, and on that December morning there was rung out the most thrilling and joyful refrain ever sung by angels or men: "Peace on earth and good will toward men." Next to Christ, the greatest Physician, and His teachings, has the profession of medicine contributed to the answer of that question. Heroes of our profession are dying every day. To-day our lives are spent in the cottage, watching over the little emaciated form, trying to bring life and health and save it to the bosom of its mother; to-morrow may be in the mansion of the rich, smoothing the pillow of the fair consumptive. There is no place in the "Hall of Fame" for even *one* of the profession of medicine, but the names of many of our profession will live forever, and their noble deeds and brilliant achievements will perpetuate their memory when the proudest monuments of earth and marble shall have crumbled away. To live in the hearts of a grateful people; to live in the affections of a mother for snatching the little one from the icy hand of death; to have the gratitude of a husband for staying the hours of death from the companion of his bosom and the mother of his children—to leave behind a record: "He went about doing good"—this is more abiding than a place on a transitory and dissolving wall of any "Hall of Fame."

Such is but a feeble portraiture of some of the achievements of medicine. Each victory over disease only leads to another. Each important discovery paves the way for another. Our work is only begun. The El Dorado of medicine is the prevention of disease. In the field of serum-therapy and the discovery of antitoxins, the greatest future of medicine lies, and why not the Louisiana State Medical Society produce a Pasteur or a Koch?

In conclusion, allow me to say, may it be the motto of every member of this Society to live up to the noble aims and high purposes of our profession. If the profession in Louisiana is to achieve great things, it must be done through an organized body. Let us rally around the banner of the Louisiana State Medical Society—let us take no backward steps. The community and State must feel and know our influence. How better can this be accomplished than the perfecting in every parish an organization from which will radiate knowledge and influence, which will tend to the upbuilding and betterment of the people. Members of the Louisiana State Medical Society and citizens of the grand commonwealth, let us endeavor to make this Society and the profession more and more necessary factors in maintaining and increasing the wealth, health, happiness and prosperity of this fair land of ours.

